# Attitude to COVID-19 Prevention With Large-Scale Social Restrictions (PSBB) in Indonesia: Partial Least Squares Structural Equation Modeling

**DOI:** 10.3389/fpubh.2020.570394

**Published:** 2020-10-30

**Authors:** Sang Gede Purnama, Dewi Susanna

**Affiliations:** ^1^Doctoral Program in Public Health, Faculty of Public Health, Universitas Indonesia, Depok, Indonesia; ^2^Department of Public Health and Preventive Medicine, Faculty of Medicine, Udayana University, Denpasar, Indonesia; ^3^Department of Environmental Health, Faculty of Public Health, Universitas Indonesia, Depok, Indonesia

**Keywords:** attitude, perception, COVID-19, Indonesia, modeling

## Abstract

There is a continuous increase in the number of COVID-19 cases in Indonesia. To control its spread, the government has implemented several strategies, such as policies associated with large-scale social restrictions (Indonesian: Pembatasan Sosial Berskala Besar or PSBB). The purpose of this study is to determine the variables that influence attitudes toward PSBB policies in Indonesia. This is a cross-sectional study with data obtained from 856 respondents from all provinces in Indonesia using the partial least squares and structural equation model (PLS-SEM). A total of 23 indicators were used to examine these policies, which were grouped into five variables: benefits of the PSBB (5 indicators), positive perception (5 indicators), negative perception (3 indicators), threatened perceptions of COVID-19 (5 indicators), and attitude toward the PSBB policy (5 indicators). The model explains over 50% of attitudes exhibited toward PSBB policy implementation and how it is influenced by the perceived benefits, negative and positive perceptions as well as the threat associated with COVID-19. The policy of stay at home, physical distancing, and always using face masks needs to be continued for the public to have a supportive attitude of the PSBB policy in preventing the transmission of COVID-19.

## Introduction

The COVID-19 pandemic, which initially started in Wuhan, China, has spread to over 200 countries worldwide. On August 12, 2020, there are 20,162,474 cases were reported, with ~737,417 deaths (1–4). In Indonesia, according to government data on August 12, 2020, there were 130,718 cases and 5,903 deaths (5).

According to the World Health Organization (WHO), the COVID-19 virus can be transmitted from an infected person to others through droplets when coughing or sneezing as well as by touching objects infected with the virus (6). The WHO recommends the mandatory use of face masks (7, 8), reducing crowds by shutting down workplaces, schools, places of worship, and other forms of social gathering. Furthermore, physical distancing needs to be maintained by staying at a distance of more than 2 m away from other people (9). Regular washing of hands, disinfecting frequently touched surfaces, and desisting from touching the mouth, nose, and eyes are also recommended (10). However, social distancing was found to be less accepted by the public than other means of control, as evidenced by a continuous increase in transmission at the local level and in communities outside the home. The Indonesian government has enacted regulation No. 21 of 2020 concerning large-scale social restrictions (PSBB) to help increase control over the spread of COVID-19.

These restrictions include closing workplaces, schools, public transportation, and socio-cultural, religious, and community activities in public places or facilities (11). The criteria for the application of PSBB are the significant and rapid increase in the number of cases and deaths from COVID-19 disease as well as epidemiological links with similar incidents in other regions or countries.

All regions in Indonesia are encouraged to implement physical and social distancing policies to prevent the spread of this virus, which is currently at the community transmission level (12, 13). Citizens in almost all the provinces in Indonesia are at risk of being infected with this virus; therefore, they are encouraged to restrict their activities.

Unfortunately, public awareness to prevent the transmission of COVID-19 is still very low; which is demonstrated by the presence of people who actively live their lives in public places. Many studies have been conducted on the attitudes and perceptions of health workers (14, 15). However, there has not been any published research regarding people's perceptions and attitudes toward PSBB policy. This study adopted a theoretical framework from the research carried out in Kenya on health workers' perceptions and attitudes toward national health care (16) to create PSBB policies. Therefore, through structural equation modeling analysis, the right variables are formed to support these policies.

## Methodology

### Conceptual Model

**Figure 1:**
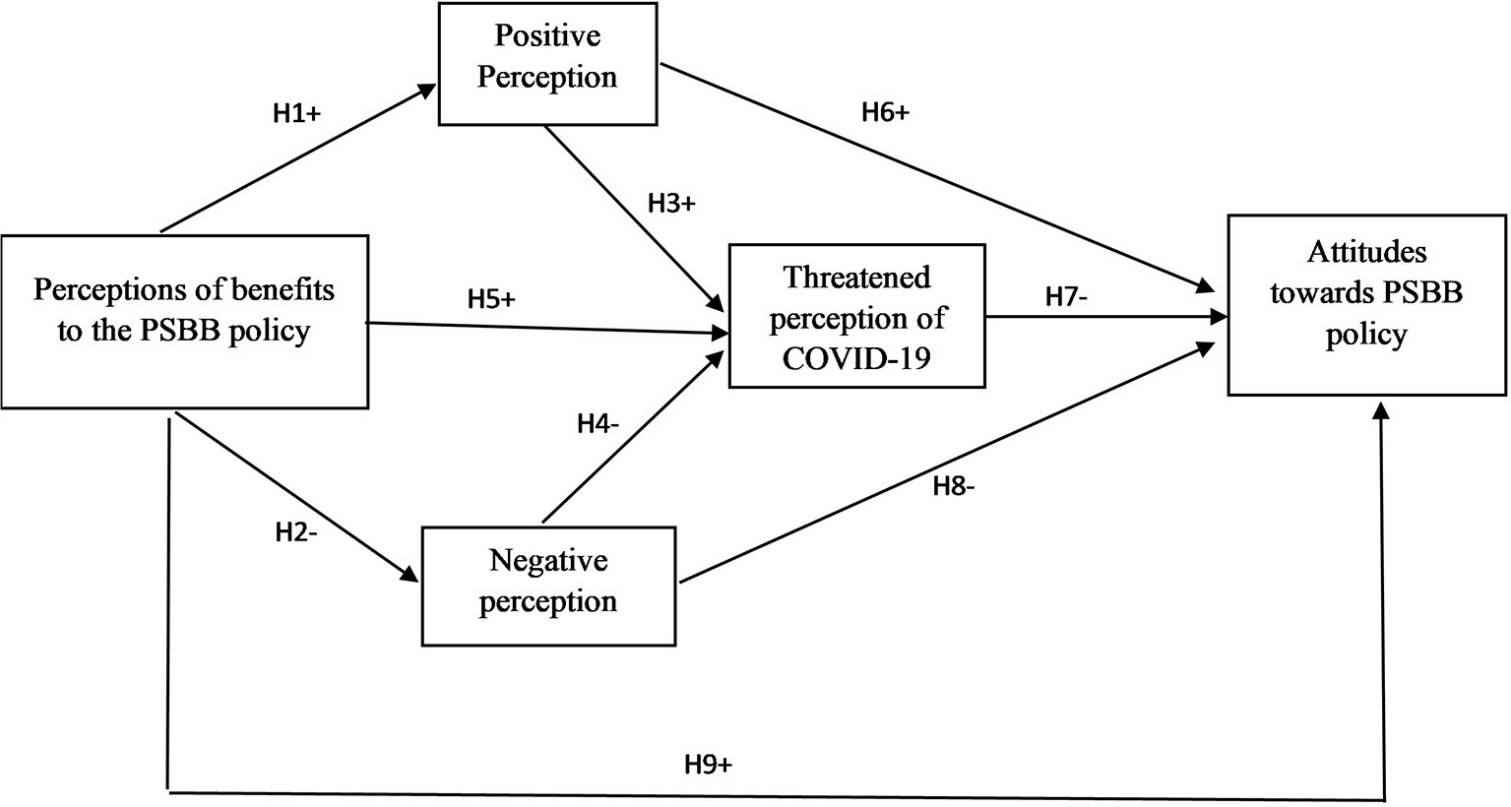
Hypothesized structural relationships of perception and attitudes toward PSBB policy.

The theoretical model adopted in this study was associated with the perceptions and attitudes of health workers in allocating national nursing resources (16) as well as perceptions and attitudes related to tourism (17, 18). The hypothesized model comprised of five latent constructs on the PSBB policy is influenced by the benefits, positive perception, negative perception, perceived threat of COVID-19, and attitude toward PSBB policy, as shown in Figure 1. The path direction represents the positive (+) and negative (–) effects of the relationship. This study examines the suitability of the model and hypothesis with SEM-PLS.

### Study Design and Data Collection

This is a cross-sectional study based on a web-based survey used to measure the five variables on attitudes toward PSBB policy influenced by the benefits, positive perception, negative perception, and perceived threat of COVID-19. Questionnaires related to the perceptions of health workers and mechanisms for national nursing resources for COVID-19 prevention were developed by the Ministry of Health (14, 16). Respondents answered these questions using a five-point Likert scale: 1 (strongly disagree), 2 (disagree), 3 (neutral), 4 (agree), and 5 (strongly agree). This tool was used because of its simplicity and ease of use. Each question item was discussed with experts to obtain the necessary suggestions and ways to further prevent the virus. The online questionnaire was tested for validity and reliability by 50 respondents, which led to a total of five invalid questions. Data were anonymously collected from respondents in 34 provinces through an online survey (19) using Google forms. It was also distributed by the Indonesian health professional organizations through WhatsApp from May 1 to May 14, 2020.

### Respondent

The respondents who participated in the online survey were above 17 years old and had resided in Indonesia for more than 6 months. They provided informed consent before filling out the questionnaire and were paid by the sponsor. A total of 868 people filled out the data, with 856 eligible responses.

Several steps were taken to prevent missing data (20). First, each respondent received an explanation of the purpose of the study by filling out documents associated with their informed consent. Second, in the web-based questionnaire survey, an automatic system was used to fill out the data, and it was discontinued when blank. Third, respondents' data were collected anonymously to ensure confidentiality. Listwise deletion was used when data were missing. Incomplete data that did not meet the requirements were not used in this study.

This study used a partial least squares (PLS-SEM) composite scheme with the SmartPLS 3.0 software to analyze four perception variables on attitudes toward PSBB policy consisting of 30 indicators. Partial least squares were used to create a structural model with the ability to map paths with many variables simultaneously. This analysis was used to predict the multicollinearity among variables (21). Table 1 shows the theoretical models proposed in this study, which examines the effect of PSBB policy influenced by the benefits, positive perception, negative perception, and perceived threat of COVID-19.

**Table 1:** Data description.

**Composite**	**Indicator**	**Definition**
Perception of benefits from PSBB	Var1a	Reduce the risk of transmitting COVID-19
	Var1b	Prevent transmission
	Var1c	Can immediately stop its transmission
	Var1d	Improves community discipline
	Var1e	Increases community participation
Positive perception	Var2a	Supports the use of masks
	Var2b	Participate in the prevention of COVID-19
	Var2c	Need to protect families from the virus
	Var2d	Support the stay at home policy
	Var2e	Support studying and working from home.
	Var2f*	Get help or facilities such as food assistance, electricity bills, given a mask from the government
Negative perception	Var3a*	Make a limited income
	Var3b	Restricting social activities outside the home
	Var3c	Not permitted to leave the area
	Var3d*	Increase in the cost for internet usage
	Var3e	Migrant workers are prohibited from returning to their hometown (mudik)
	Var3f*	Basic needs become limited and expensive
Threatened perception of COVID-19	Var5a	Fear of being infected
	Var5b	Feeling afraid that foreign guests are coming with COVID-19
	Var5c	Fear of family members contracting the virus
	Var5d	Fear a family member died because of COVID-19
	Var5e	Scared of leaving the house
	Var5f*	Feeling anxious on news related to the virus
	Var5g*	Scared of the sanctions associated with violating the policy
Attitudes toward PSBB policy	Var4a	Participate in the socialization of PSBB policies
	Var4b	Stay at home
	Var4c*	Work from home
	Var4d	Reduce social activities
	Var4e	Physical distancing
	Var4f	Migrant workers were not allowed to return to their hometowns

* *These indicators were not included in latent variables due to the multicollinearity criteria of PLS-SEM*.

### Measurement of Variables

This study consists of five variables measuring 30 indicators using a Likert scale of 1 (strongly disagree), 2 (disagree), 3 (neutral), 4 (agree), and 5 (strongly agree). The following constructs are part of this model:

The attitude toward the PSBB policy is the dependent variable, which means that the respondent's attitude is carried out in daily life. It is measured by six indicators, consisting of those who participate in the socialization of PSBB policies: stay at home, work from home, reduce social activities, physical distancing, and migrant workers who do not return to their hometown or village during or before major holidays (mudik).

The perception of benefits from the PSBB policy is associated with assessing the policy implemented by the government that benefits the community. It consists of six indicators that reduce the risk of transmitting COVID-19 and prevent its spread, thereby improving community discipline and participation.

Positive perception is associated with respondent's assessments of the PSBB policy, which is in line with the expectations of its regulations. It comprises of six indicators: support the use of masks, participate in preventing COVID-19, protecting families from the virus, not leaving the house, studying and working from home, and getting help or convenience such as food and bills assistance, and masks from the government.

Negative perception is the respondent's assessment of PSBB policies that are not in line with their expectations. It consists of six indicators: receiving limited income, restricting social activities outside the home, not permitted to leave the area, increasing cost for internet usage, migrant workers are prohibited from returning to their hometown (mudik), and basic needs become limited and expensive.

Threatened perception of COVID-19 frightens respondents. It consists of seven indicators, namely fear of being infected, increased by foreign guests, family members contracting the virus, fear of the death of a family, scared of leaving the house, feeling anxious about news related to the virus, and scared of the sanctions.

### Statistical Procedure

Structural equation models are analyzed in two stages: measurement and structural model analyses (22). The first stage describes the model being measured by connecting the constructs and indicators according to the theory. After obtaining the quality of the measured data, a structural model is used to determine the relationship between the construction or hypothesis model. This is carried out to make valid and reliable measurement scales to prove the structural model hypothesis. This study used the Smart-PLS 3.2.7. software.

### Ethical Approval

The study's ethical clearance was obtained from the Public Health Faculty, Universitas Indonesia (No. 198/UN2.F10. D11/PPM.00.02/2020). This study was carried out in accordance with the Declaration of Helsinki and the recommendations of those committees with written informed consent from all participants.

## Results

The respondents in the provinces were distributed as follows: Bali (21.3%), West Java (14.9%), East Java (10%), South Sulawesi (7.8%), Riau (7.7%), and Central Java (5.6%) (Table 2). The Java-Bali region had a relatively high trend of increasing cases compared to other provinces, due to the population density and higher mobility. The demographic characteristics of the 856 respondents were aged 17–24 years (39%). The highest gender distribution, education level, and employment type were male (70.8%), secondary education (55%), and students (28.6%), respectively. Furthermore, the health workers and government officers were 15.2 and 14.7%, respectively.

**Table 2:** Sociodemographic characteristics of respondents.

**Province**	**Frequency (*N* = 856)**	**Percent**
Bali	183	21.38
South Sulawesi	67	7.83
Riau	66	7.71
West Nusa Tenggara	61	7.13
East Java	86	10.05
Central Java	48	5.61
West Java	128	14.95
DI Yogyakarta	12	1.40
DKI Jakarta	65	7.59
Bengkulu	32	3.74
Banten	21	2.45
South Sumatra	13	1.52
North Sumatra	9	1.05
West Sumatra	7	0.82
Nanggroe Aceh Darussalam	5	0.58
Lampung	15	1.75
Kepulauan Riau	9	1.05
East Kalimantan	12	1.40
South Kalimantan	8	0.93
West Papua	3	0.35
East Nusa Tenggara	3	0.35
Sulawesi Tenggara	3	0.35
Age (years)		
17–24	334	39
25–29	146	17.1
30–34	115	13.4
35–39	89	10.4
40–44	69	8.1
45–49	42	4.9
50–54	39	4.6
55–59	16	1.9
60+	6	0.7
Gender		
Male	606	70.8
Female	250	29.2
Education		
Without education	7	0.8
Primary education	16	1.9
Secondary education	471	55.0
Diploma	145	16.9
Bachelor's degree	172	20.1
Higher than bachelor's degree	45	5.3
Occupation		
Government officer	126	14.7
Health workers	130	15.2
Lecturer	23	2.7
Teacher	7	0.8
Housewife	56	6.5
College student	40	4.7
Student	245	28.6
Industrial staff	141	16.5
Self-employed	55	6.4
Others	33	3.9

### Measurement Model

#### Composite Mode A

**Figure 2:**
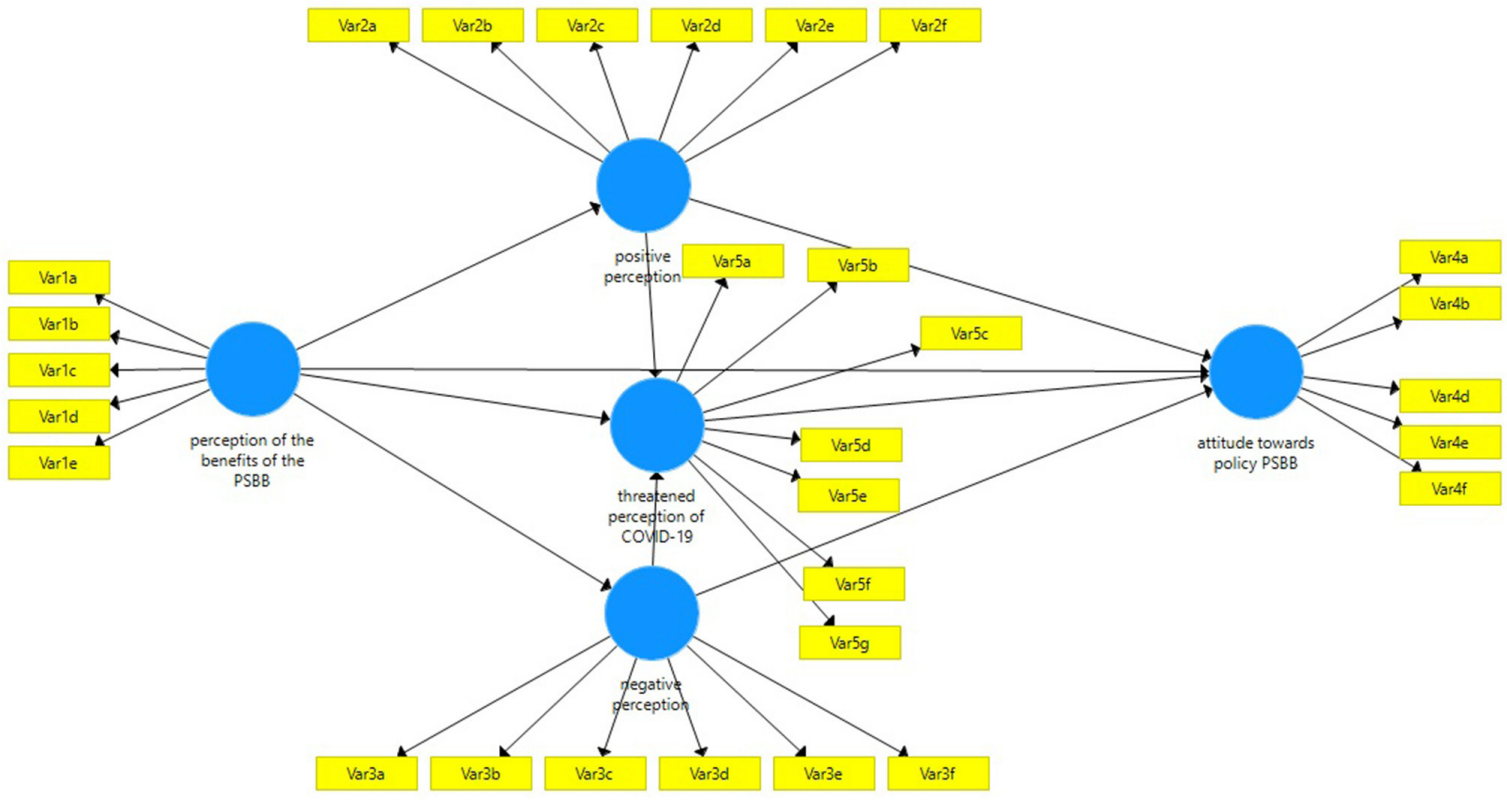
Research model.

The composite measurement model in mode A was assessed in terms of individual item reliability, discriminant validity, convergent validity, and construct reliability, which were analyzed using the through-loading factors shown in Figure 2.

Composite reliability is more precise than internal consistency. It can be used with PLS-SEM to accommodate different loading indicators. Validity assessment was carried out by calculating convergent and discriminant validities. Table 3 illustrates the cutoff value of 0.7 for 3 measurements, namely Cronbach's alpha, Dijkstra-Henseler's rho coefficients, and composite reliability. The third convergent validity is proven because each construct's average variance extracted (AVE) is higher than 0.5. Table 3 shows that the measurement model fits the criteria.

**Table 3:** Validity and reliability measurement.

**Composite**	**Cronbach's alpha**	**Dijikstra-Henseler's rho**	**Composite reliability (CR)**	**Average variance extracted (AVE)**
Attitude toward PSBB policy	0.865	0.774	0.899	0.599

Table 4 presents the results of discriminant validity through the Heterotrait-Monotrait (HTMT) correlation ratio. All constructs are in accordance with the discriminant validity because the confidence interval does not contain a zero value. This means that each variable is different from one another.

**Table 4:** HTMT inference.

HTMT inference*	**Original sample**	**Sample mean**	**5%**	**95%**
Attitude toward PSBB➔benefit	0.534	0.533	0.445	0.609
Attitude toward PSBB➔feel threatened	0.479	0.478	0.372	0.576
Attitude toward PSBB➔positive perception	0.765	0.765	0.683	0.837
Attitude toward PSBB➔negative perception	0.504	0.506	0.407	0.619

* *Significance, the 95% confidence interval can be corrected using the bootstrap procedure with 10,000 replications*.

*HTMT, Heterotrait–Monotrait*.

The data examined above in the measurement model show that the construct is reliable and valid.

#### Composite Mode B

**Figure 3:**
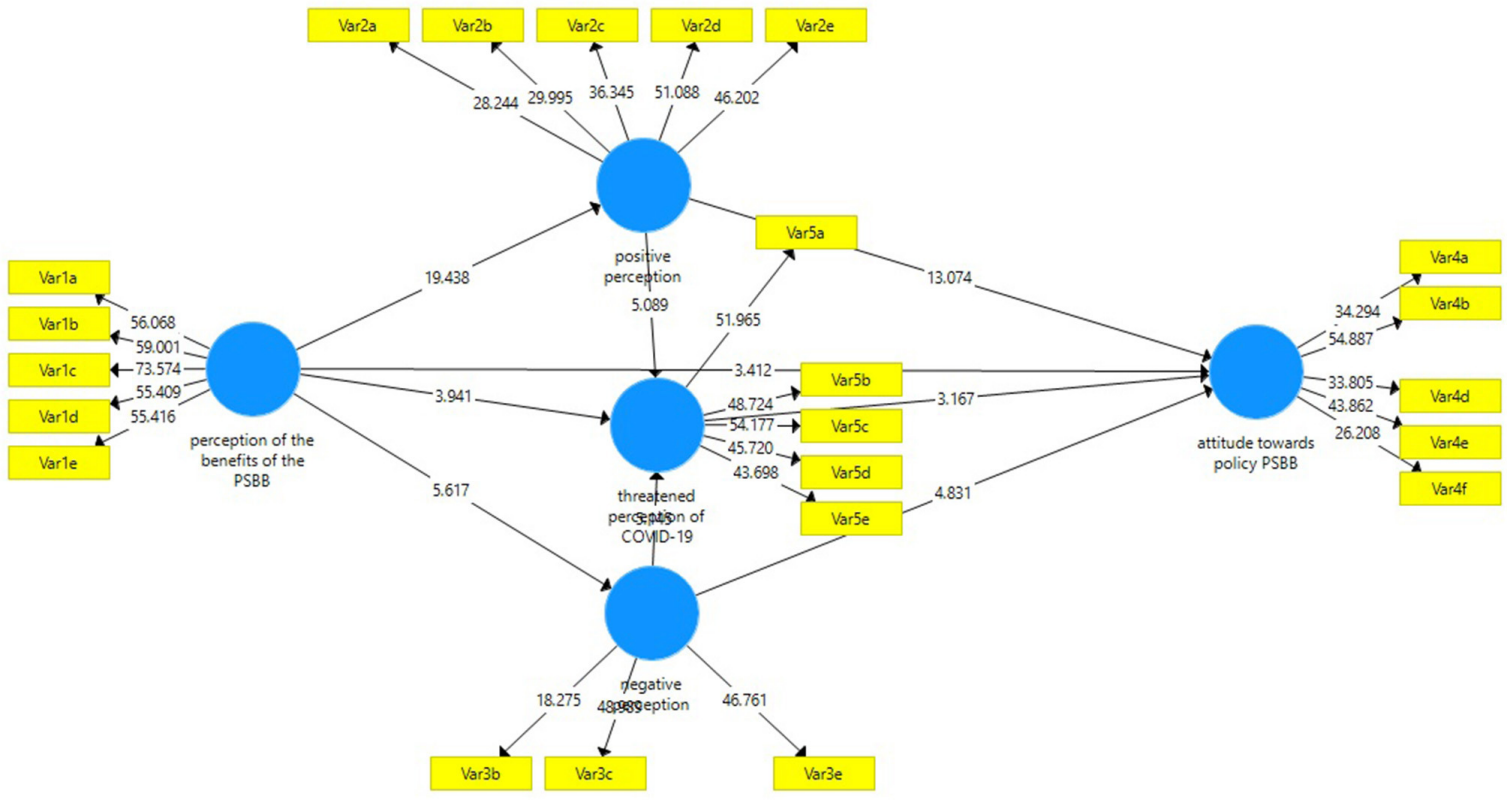
SEM-PLS results model.

The composite measurement model in mode B was assessed in terms of the collinearity between the indicators, significance, and relevance of the external weights. First, this was carried out by removing the indicator when it exceeded the value of the impact factor variance (VIF = 3). As a result of this process, only the indicators shown in Table 1 are not collinear. Second, the relevance of weights was analyzed, as shown in Figure 3, with the relevance of indicators in construction for latent variables. Finally, 10,000 subsamples were used to start bootstrapping, and to determine the ability to the outside weight significantly different from zero. Indicators with weights were insignificant, with a significant loading of 0.50 above the relevant values, as shown in Table 5.

**Table 5:** Significance of weights.

	Original sample (O)*	**t**	**Loading**	**Lo95**	**Hi95**
**Perception of benefits**					
Var1a	0.248	24.965	0.855	0.228	0.268
Var1b	0.252	25.275	0.869	0.230	0.270
Var1c	0.224	26.016	0.851	0.207	0.241
Var1d	0.226	22.641	0.831	0.207	0.247
Var1e	0.230	23.058	0.828	0.212	0.250
**Positive perception**					
Var2a	0.224	22.448	0.758	0.204	0.242
Var2b	0.244	22.707	0.739	0.225	0.265
Var2c	0.226	25.446	0.808	0.209	0.243
Var2d	0.280	25.114	0.833	0.259	0.304
Var2e	0.289	23.723	0.809	0.266	0.314
**Negative perception**					
Var3b	0.273	7.996	0.725	0.197	0.331
Var3c	0.433	17.938	0.879	0.386	0.480
Var3e	0.493	15.675	0.855	0.436	0.559
**Attitude toward PSBB policy**					
Var4a	0.251	21.793	0.759	0.229	0.275
Var4b	0.273	28.682	0.856	0.255	0.292
Var4d	0.232	24.450	0.803	0.213	0.251
Var4e	0.260	23.316	0.829	0.239	0.282
Var4f	0.226	22.431	0.768	0.207	0.247
**Threatened perception of COVID-19**					
Var5a	0.229	18.817	0.839	0.206	0.253
Var5b	0.232	20.270	0.845	0.209	0.255
Var5c	0.248	19.655	0.862	0.221	0.270
Var5d	0.234	17.452	0.841	0.205	0.259
Var5e	0.262	13.899	0.773	0.229	0.300

* *Significance in p < 0.001, t statistic, and 95% bias-corrected confidence interval performed by a bootstrapping procedure with 10,000 replications*.

### Structural Model

After verifying the appropriate values of the construction measurements, an assessment of the structural model was carried out using 10,000 resampling bootstraps. The path coefficients and the significance level of their 10,000 resampling bootstraps are reported in Table 6 and Figure 3. Furthermore, Table 6 also shows that the VIF construction ranges from 1,000 to 1,700, indicating that there is no collinearity between variables. This study also assesses quality by examining whether the predictive relevance of the whole model has a *Q*^2^-value above zero; therefore, it fits in the model predictions. The coefficient of determination (*R*^2^) also exceeds 0.1 for endogenous latent variables. Therefore, the construct has an acceptable predictive power quality.

**Table 6:** Whole sample results.

	**Path**	***t***	***p***	**Lo95**	**Hi95**	**f^**2**^**	**VIF**
**Direct effect**							
Benefit➔positive perception	0.550	19.012	0.000	0.493	0.607	0.434	1.000
	*R*^2^ **=** **0.302**						
Benefit➔negative perception	0.221	5.493	0.000	0.151	0.303	0.051	1.000
	*R*^2^ **=** **0.048**						
Positive perception➔threatened perception	0.226	5.113	0.000	0.145	0.312	0.043	1.497
Negative perception➔threatened perception	0.204	5.343	0.000	0.139	0.288	0.048	1.098
Benefit➔threatened perception	0.179	3.981	0.000	0.095	0.269	0.028	1.443
	*R*^2^ **=** **0.210**						
Benefit➔attitude toward PSBB policy	0.109	3.471	0.001	0.047	0.170	0.017	1.484
Threatened perception➔attitude toward PSBB policy	0.124	3.224	0.001	0.046	0.191	0.026	1.270
Positive perception➔attitude toward PSBB policy	0.497	12.464	0.000	0.414	0.576	0.336	1.562
Negative perception➔attitude toward PSBB policy	0.219	4.888	0.000	0.137	0.316	0.089	1.150
	*R*^2^ **=** **0.529**, ***Q***^**2**^ **=** **0.527**						
**Indirect effect**						VAF	
Benefit➔perception +➔threatened perception	0.170	5.787	0.000	0.118	0.233	20.8	na
Benefit➔perception +➔threatened perception attitude toward PSBB policy	0.365	12.712	0.000	0.311	0.424	15.3	na
Perception positive➔threatened perception➔attitude toward PSBB policy	0.028	2.585	0.010	0.008	0.048	7.6	na
Perception negative➔threatened perception➔attitude toward PSBB policy	0.025	2.474	0.013	0.008	0.044	5.2	na

*ns, not significant. Significance, t statistics, and 95% confidence can be corrected. A bootstrap procedure performs the interval with 10,000 replications. VIF, Inflation of model variants in factors; VAF, variance recorded*.

Table 6 shows that the PSBB policy influenced by the benefits, positive, negative, and perceived threat of COVID-19 directly influence community attitudes (*p* values <0.001 and 0.001). Furthermore, each variable has a positive relationship with attitude, and the indirect effect can be seen from the value of VAF, which indicates that the proportion mediated by the total effect of feeling threatened through negative perception is 7.6%, as shown in the indirect effect in Table 6. This model explains that the benefits, positive, and negative perceptions influence 52.9% of attitudes toward PSBB policy, and the perceived threat of COVID-19.

## Discussion

In this cross-sectional study (23), PLS-SEM was used to explore the determinant relationships of perceptions and attitudes toward COVID-19 policy in Indonesia. The general population in several provinces in Indonesia were used to represent the conditions in each region. The PSBB policy is not carried out simultaneously in all regions, although all of them have the potential risk of COVID-19 transmission.

The study analyzed a total of five variables with 30 indicators that influence attitudes toward the policies of PSBB. After analysis using PLS-SEM, 23 indicators were obtained. This model explains that attitudes toward PSBB policy are influenced by the benefits, positive and negative perceptions, and perceived threat of COVID-19.

Policymakers need to understand how to prevent the transmission of COVID-19 as opening public access to infrastructures without considering epidemiological studies can lead to rapid transmission (24). This study describes the attitudes of the community in supporting large-scale social restriction policies carried out in Indonesia. A positive attitude toward this policy causes the community to comply with the regulations willingly and understand the benefits.

Most of the survey respondents were located on Java Island. The distribution of respondents does not represent all provinces in Indonesia; however, Java Island is densely populated, and high population density is one of the risks in the spread of COVID-19 (25). In particular, the challenges are greater in limiting population mobility and social distancing (26). Studies in China also show that people infected with COVID-19 tend to be in densely populated areas (27). A study in Brazil also found that air transportation, population density, and temperature affect the spread of COVID-19 (28).

Most of the respondents in the survey were male and aged 17–24 years. The PSBB policy encouraged school closure and home study through electronic media and online applications. Students' perceptions of the benefits and positive attitude toward PSBB tend to influence their compliance with PSBB policies. Efforts are needed to increase student awareness in preventing the transmission of COVID-19 (29). School closure policies must also be supported by strict social distancing policies (11).

Through a cross-sectional approach, PLS-SEM analysis can be carried out quickly in the general population. This model is designed to determine the respondent's attitude toward the PSBB policy at a specific time. However, it is necessary to carry out further research with a longitudinal approach to determine the comparison of changes in people's attitudes toward the PSBB policy according to the observation period.

The community's attitude as a dependent variable is associated with participating in socializing PSBB, staying at home, reducing social activities, maintaining a safe distance from others, and migrant workers not returning to their hometown. This is consistent with the WHO recommendation to prevent transmission of COVID-19 on staying at home and maintaining a safe distance of more than 2 m (30). These measures aim to stop the spread of the virus, which is transmitted from an infected person to another through droplets when coughing (31–33).

The city of Wuhan in China was locked down to prevent the rapid transmission of the virus (34). This policy was implemented under strict action, discipline, and punishment for violators, and food was provided for the population. However, in Indonesia, restrictions were placed on community activities such as schools, workplaces, and religious places, with access to markets and population logistics. The government also failed to cover the daily needs of the population and hopes that the transmission rate will be reduced through the PSBB policy.

The PSBB policy reduces the risk of COVID-19 transmission by preventing its spread and stopping its transmission through increased community discipline and involvement. The government has supported socialization through electronic platforms and social media, with teachers and community leaders providing adequate information on the benefits of the PSBB policy. It is expected that the public complies with this regulation due to the increase in public awareness.

Furthermore, increased understanding of the community causes positive attitudes such as supporting the use of masks, wanting to protect families from contracting the virus, supporting the idea of not leaving the house as well as learning, and working from home, and participating in the prevention of COVID-19 (35–37). Health education needs to be given to vulnerable populations infected with COVID-19, such as the elderly (38, 39) used to avoid stress (40). The use of masks is an easy, cheap, and effective way to prevent transmission (41); therefore, the WHO recommends its usage (42–44).

The negative perceptions toward the PSBB policy are limited social activities outside the home and not being allowed to leave the area or town. Therefore, traders, construction, and factory workers lost their income negatively affecting their socioeconomic status. Furthermore, those who have family outside their area were also prohibited from traveling. This makes the population uncomfortable with the PSBB policy, thereby leading to anxiety, lack of sleep, depression (16–28%), and stress (8%) (45).

The perception of being threatened with COVID-19 consists of a feeling of fear of being exposed to the virus, foreign guests coming into the country infected, and fear of leaving the house (46–48). It is also associated with the fear of family members being infected with the virus and the possibility of death. These perceptions encourage people to take the necessary preventive steps not leaving the house and adhering to the government's recommendation to conduct a PSBB.

### Study Strengths and Limitations

The strengths of this research were determined by measuring the benefits, positive, negative, and perceived threat of COVID-19 in accordance with the use of PSBB. These perceptions can influence respondents' attitudes toward the PSBB policy. This model is usually used in social research in tourism as well as in the health sector (16–18, 49).

The PLS-SEM was chosen for component-based social research with formative construct properties. This approach is variant based and has the ability to estimate composites and factors (50, 51). It is also useful to predict the dependent variable within a large number of independent variables. In addition, through this approach, an appropriate structural equation model can be made with variables related to attitudes toward PSBB policy.

This study is limited by the use of online surveys, which are provided by the general public rather than specific targets. However, with the general public, there are various advantages, such as the ability to reach all groups in a broad range, depending on the PSBB policy. However, this situation was not uniform in all places, which had a non-concurrent implementation process.

### Policy Implications and Future Research

This study is useful for policymakers, especially for health interventions and health education programs in efforts to control COVID-19. The PSBB policy with restrictions on community activities and entering and leaving the area. Schools and workplaces are closed, but learning activities can be carried out online. The PSBB is also supported by tracking and finding people who are exposed (52). Efforts to control COVID-19 in other countries by limiting community activities at the beginning of the pandemic, such as lockdown implementations, have proven effective in reducing the transmission of the virus (53, 54). However, this approach has been reported to have an impact on psychological and economic factors in society (55–57).

The results of this study have implications for controlling COVID-19 in various regions, particularly in Indonesia. The public needs to obtain adequate educational awareness on the perception and benefits of the PSBB policy, which can positively impact the public's attitudes to abide by the policy. This study contributes to the addition of academic literature by applying the PLS-SEM to explore the relationship between attitudes toward PSBB and COVID-19 spread. Subsequent studies can be conducted in certain areas to control numerous factors by the timing of a particular PSBB implementation, to ensure that the impact is clear when compared to many regions with a non-concurrent PSBB period.

## Conclusion

The PSBB policy needed to obtain adequate attention from the community to prevent the rapid transmission of COVID-19 in Indonesia. Furthermore, the attitude of those that support this policy tends to affect the successful implementation of this program. This model explains that 52.9% of attitudes toward PSBB policies are influenced by perceptions of the benefits of the PSBB policy, positive perceptions, negative perceptions, and perceptions of the threat of COVID-19. The policy of not leaving the house, keeping a safe distance, and always using face masks needs to be continued for the public to support the PSBB policy in preventing further transmission.

## Data Availability Statement

Derived data supporting the findings of this study are available from the corresponding author on request. Requests to access the datasets should be directed to Sang Gede Purnama, sangpurnama@unud.ac.id.

## Ethics Statement

The studies involving human participants were reviewed and approved by Public Health Faculty, Universitas Indonesia. The patients/participants provided their written informed consent to participate in this study. Written informed consent was obtained from the individual(s) for the publication of any potentially identifiable images or data included in this article.

## Author Contributions

SP developed a draft proposal, study design, collected data, and revised the results. Meanwhile, the DS participants made study drafts, researched designs, collected data, and revised the results. All authors contributed to the article and approved the submitted version.

## Conflict of Interest

The authors declare that the research was conducted in the absence of any commercial or financial relationships that could be construed as a potential conflict of interest.
